# Supplementary material for: Heparin-based hydrogel scaffolding alters the transcriptomic profile and increases the chemoresistance of MDA-MB-231 triple-negative breast cancer cells
Source: Biomater Sci. 2020 Feb 13;8(10):2786–96. doi: 10.1039/c9bm01481k (PMC7497406; doi:10.1039/c9bm01481k)
Supplement: Supplementary file 2 [file BM-008-C9BM01481K-s002.zip › Supplementary File 4/EGFvControl/Pathways/my_analysis.Gsea.1545200981068/HALLMARK_E2F_TARGETS.html]

Details for gene set HALLMARK\_E2F\_TARGETS[GSEA]

|  || Dataset | expr.class.cls#EGF\_versus\_CONTROL.class.cls#EGF\_versus\_CONTROL\_repos |
| Phenotype | class.cls#EGF\_versus\_CONTROL\_repos |
| Upregulated in class | EGF |
| GeneSet | HALLMARK\_E2F\_TARGETS |
| Enrichment Score (ES) | 0.6842201 |
| Normalized Enrichment Score (NES) | 3.1536756 |
| Nominal p-value | 0.0 |
| FDR q-value | 0.0 |
| FWER p-Value | 0.0 |
Table: GSEA Results Summary

  

Fig 1: Enrichment plot: HALLMARK\_E2F\_TARGETS      
 Profile of the Running ES Score & Positions of GeneSet Members on the Rank Ordered List

  

| PROBE | DESCRIPTION (from dataset) | GENE SYMBOL | GENE\_TITLE | RANK IN GENE LIST | RANK METRIC SCORE | RUNNING ES | CORE ENRICHMENT || 1 | CENPM | na |  |  | 32 | 2.800 | 0.0106 | Yes |
| 2 | TCF19 | na |  |  | 94 | 2.369 | 0.0178 | Yes |
| 3 | CDC25A | na |  |  | 107 | 2.312 | 0.0273 | Yes |
| 4 | AURKA | na |  |  | 163 | 2.181 | 0.0340 | Yes |
| 5 | MTHFD2 | na |  |  | 172 | 2.163 | 0.0431 | Yes |
| 6 | EZH2 | na |  |  | 227 | 2.053 | 0.0492 | Yes |
| 7 | PRPS1 | na |  |  | 267 | 2.008 | 0.0560 | Yes |
| 8 | CDCA8 | na |  |  | 295 | 1.962 | 0.0632 | Yes |
| 9 | AURKB | na |  |  | 328 | 1.916 | 0.0699 | Yes |
| 10 | ASF1A | na |  |  | 333 | 1.907 | 0.0781 | Yes |
| 11 | UNG | na |  |  | 348 | 1.892 | 0.0857 | Yes |
| 12 | PHF5A | na |  |  | 357 | 1.884 | 0.0935 | Yes |
| 13 | TFRC | na |  |  | 359 | 1.882 | 0.1017 | Yes |
| 14 | KIF18B | na |  |  | 382 | 1.859 | 0.1087 | Yes |
| 15 | CCNE1 | na |  |  | 408 | 1.841 | 0.1155 | Yes |
| 16 | NOLC1 | na |  |  | 443 | 1.809 | 0.1216 | Yes |
| 17 | DSCC1 | na |  |  | 459 | 1.796 | 0.1287 | Yes |
| 18 | RAD51C | na |  |  | 510 | 1.767 | 0.1339 | Yes |
| 19 | SMC6 | na |  |  | 525 | 1.756 | 0.1408 | Yes |
| 20 | CDKN3 | na |  |  | 562 | 1.729 | 0.1465 | Yes |
| 21 | CDC20 | na |  |  | 590 | 1.712 | 0.1526 | Yes |
| 22 | SUV39H1 | na |  |  | 628 | 1.686 | 0.1581 | Yes |
| 23 | CDKN2C | na |  |  | 635 | 1.679 | 0.1651 | Yes |
| 24 | PLK4 | na |  |  | 650 | 1.670 | 0.1717 | Yes |
| 25 | RAD51AP1 | na |  |  | 662 | 1.664 | 0.1785 | Yes |
| 26 | ATAD2 | na |  |  | 667 | 1.662 | 0.1856 | Yes |
| 27 | CDCA3 | na |  |  | 687 | 1.652 | 0.1918 | Yes |
| 28 | DDX39A | na |  |  | 731 | 1.621 | 0.1967 | Yes |
| 29 | DNMT1 | na |  |  | 732 | 1.621 | 0.2038 | Yes |
| 30 | TK1 | na |  |  | 759 | 1.605 | 0.2095 | Yes |
| 31 | POLE | na |  |  | 760 | 1.605 | 0.2165 | Yes |
| 32 | NME1 | na |  |  | 764 | 1.603 | 0.2234 | Yes |
| 33 | RFC2 | na |  |  | 818 | 1.582 | 0.2275 | Yes |
| 34 | PRIM2 | na |  |  | 835 | 1.575 | 0.2336 | Yes |
| 35 | PA2G4 | na |  |  | 838 | 1.574 | 0.2404 | Yes |
| 36 | HELLS | na |  |  | 862 | 1.566 | 0.2461 | Yes |
| 37 | SRSF1 | na |  |  | 863 | 1.566 | 0.2530 | Yes |
| 38 | RRM2 | na |  |  | 890 | 1.557 | 0.2584 | Yes |
| 39 | E2F8 | na |  |  | 891 | 1.556 | 0.2653 | Yes |
| 40 | MCM2 | na |  |  | 906 | 1.550 | 0.2713 | Yes |
| 41 | SNRPB | na |  |  | 933 | 1.538 | 0.2767 | Yes |
| 42 | UBR7 | na |  |  | 941 | 1.533 | 0.2831 | Yes |
| 43 | UBE2T | na |  |  | 949 | 1.531 | 0.2894 | Yes |
| 44 | GINS1 | na |  |  | 968 | 1.523 | 0.2952 | Yes |
| 45 | PLK1 | na |  |  | 1074 | 1.488 | 0.2962 | Yes |
| 46 | NUP205 | na |  |  | 1076 | 1.487 | 0.3026 | Yes |
| 47 | MCM7 | na |  |  | 1099 | 1.479 | 0.3080 | Yes |
| 48 | PRKDC | na |  |  | 1101 | 1.479 | 0.3144 | Yes |
| 49 | DCTPP1 | na |  |  | 1111 | 1.474 | 0.3204 | Yes |
| 50 | RAN | na |  |  | 1116 | 1.473 | 0.3267 | Yes |
| 51 | TRIP13 | na |  |  | 1123 | 1.471 | 0.3328 | Yes |
| 52 | NOP56 | na |  |  | 1153 | 1.459 | 0.3377 | Yes |
| 53 | GINS3 | na |  |  | 1175 | 1.449 | 0.3429 | Yes |
| 54 | HMGB2 | na |  |  | 1183 | 1.446 | 0.3489 | Yes |
| 55 | TACC3 | na |  |  | 1207 | 1.439 | 0.3540 | Yes |
| 56 | NUDT21 | na |  |  | 1211 | 1.437 | 0.3602 | Yes |
| 57 | USP1 | na |  |  | 1270 | 1.423 | 0.3634 | Yes |
| 58 | DLGAP5 | na |  |  | 1276 | 1.420 | 0.3694 | Yes |
| 59 | RACGAP1 | na |  |  | 1310 | 1.409 | 0.3738 | Yes |
| 60 | BRCA2 | na |  |  | 1333 | 1.399 | 0.3788 | Yes |
| 61 | RFC3 | na |  |  | 1349 | 1.393 | 0.3841 | Yes |
| 62 | HMMR | na |  |  | 1351 | 1.393 | 0.3902 | Yes |
| 63 | RPA1 | na |  |  | 1382 | 1.384 | 0.3947 | Yes |
| 64 | SMC4 | na |  |  | 1418 | 1.372 | 0.3988 | Yes |
| 65 | TRA2B | na |  |  | 1432 | 1.369 | 0.4042 | Yes |
| 66 | LMNB1 | na |  |  | 1433 | 1.368 | 0.4102 | Yes |
| 67 | EXOSC8 | na |  |  | 1440 | 1.366 | 0.4159 | Yes |
| 68 | BIRC5 | na |  |  | 1448 | 1.364 | 0.4215 | Yes |
| 69 | NBN | na |  |  | 1465 | 1.358 | 0.4266 | Yes |
| 70 | MELK | na |  |  | 1529 | 1.339 | 0.4292 | Yes |
| 71 | ZW10 | na |  |  | 1550 | 1.334 | 0.4340 | Yes |
| 72 | AK2 | na |  |  | 1577 | 1.326 | 0.4384 | Yes |
| 73 | MCM4 | na |  |  | 1617 | 1.313 | 0.4421 | Yes |
| 74 | BRCA1 | na |  |  | 1620 | 1.312 | 0.4478 | Yes |
| 75 | PAICS | na |  |  | 1636 | 1.310 | 0.4527 | Yes |
| 76 | MCM6 | na |  |  | 1672 | 1.301 | 0.4566 | Yes |
| 77 | MAD2L1 | na |  |  | 1695 | 1.296 | 0.4611 | Yes |
| 78 | SPAG5 | na |  |  | 1703 | 1.294 | 0.4665 | Yes |
| 79 | PCNA | na |  |  | 1712 | 1.291 | 0.4717 | Yes |
| 80 | KIF4A | na |  |  | 1715 | 1.291 | 0.4773 | Yes |
| 81 | PPP1R8 | na |  |  | 1734 | 1.285 | 0.4820 | Yes |
| 82 | NCAPD2 | na |  |  | 1768 | 1.276 | 0.4858 | Yes |
| 83 | BRMS1L | na |  |  | 1784 | 1.272 | 0.4906 | Yes |
| 84 | TIMELESS | na |  |  | 1795 | 1.269 | 0.4957 | Yes |
| 85 | ING3 | na |  |  | 1800 | 1.268 | 0.5010 | Yes |
| 86 | DIAPH3 | na |  |  | 1832 | 1.260 | 0.5049 | Yes |
| 87 | SRSF2 | na |  |  | 1835 | 1.260 | 0.5104 | Yes |
| 88 | XPO1 | na |  |  | 1845 | 1.259 | 0.5154 | Yes |
| 89 | TIPIN | na |  |  | 1860 | 1.254 | 0.5202 | Yes |
| 90 | IPO7 | na |  |  | 1923 | 1.241 | 0.5224 | Yes |
| 91 | KPNA2 | na |  |  | 1925 | 1.241 | 0.5278 | Yes |
| 92 | LBR | na |  |  | 1968 | 1.230 | 0.5309 | Yes |
| 93 | KIF2C | na |  |  | 1975 | 1.227 | 0.5360 | Yes |
| 94 | XRCC6 | na |  |  | 1990 | 1.222 | 0.5406 | Yes |
| 95 | STAG1 | na |  |  | 2035 | 1.212 | 0.5437 | Yes |
| 96 | CSE1L | na |  |  | 2038 | 1.211 | 0.5489 | Yes |
| 97 | BARD1 | na |  |  | 2041 | 1.211 | 0.5541 | Yes |
| 98 | CKS1B | na |  |  | 2048 | 1.207 | 0.5591 | Yes |
| 99 | RBBP7 | na |  |  | 2055 | 1.207 | 0.5640 | Yes |
| 100 | TMPO | na |  |  | 2091 | 1.199 | 0.5675 | Yes |
| 101 | TOP2A | na |  |  | 2151 | 1.189 | 0.5696 | Yes |
| 102 | MCM3 | na |  |  | 2175 | 1.183 | 0.5736 | Yes |
| 103 | CENPE | na |  |  | 2189 | 1.181 | 0.5781 | Yes |
| 104 | NUP107 | na |  |  | 2219 | 1.174 | 0.5817 | Yes |
| 105 | ANP32E | na |  |  | 2263 | 1.164 | 0.5845 | Yes |
| 106 | CCP110 | na |  |  | 2292 | 1.159 | 0.5881 | Yes |
| 107 | ORC6 | na |  |  | 2344 | 1.146 | 0.5905 | Yes |
| 108 | NUP153 | na |  |  | 2368 | 1.140 | 0.5943 | Yes |
| 109 | SSRP1 | na |  |  | 2378 | 1.137 | 0.5988 | Yes |
| 110 | LYAR | na |  |  | 2416 | 1.129 | 0.6018 | Yes |
| 111 | TBRG4 | na |  |  | 2442 | 1.125 | 0.6054 | Yes |
| 112 | KIF22 | na |  |  | 2457 | 1.122 | 0.6096 | Yes |
| 113 | DONSON | na |  |  | 2514 | 1.109 | 0.6115 | Yes |
| 114 | POLA2 | na |  |  | 2529 | 1.105 | 0.6157 | Yes |
| 115 | DEPDC1 | na |  |  | 2541 | 1.104 | 0.6199 | Yes |
| 116 | POLE4 | na |  |  | 2559 | 1.100 | 0.6239 | Yes |
| 117 | ESPL1 | na |  |  | 2577 | 1.097 | 0.6278 | Yes |
| 118 | RNASEH2A | na |  |  | 2596 | 1.095 | 0.6316 | Yes |
| 119 | POLD1 | na |  |  | 2630 | 1.089 | 0.6347 | Yes |
| 120 | GINS4 | na |  |  | 2641 | 1.087 | 0.6389 | Yes |
| 121 | UBE2S | na |  |  | 2671 | 1.081 | 0.6422 | Yes |
| 122 | ORC2 | na |  |  | 2772 | 1.065 | 0.6416 | Yes |
| 123 | EIF2S1 | na |  |  | 2819 | 1.058 | 0.6438 | Yes |
| 124 | PNN | na |  |  | 2841 | 1.054 | 0.6473 | Yes |
| 125 | DEK | na |  |  | 2886 | 1.046 | 0.6496 | Yes |
| 126 | RANBP1 | na |  |  | 2912 | 1.042 | 0.6528 | Yes |
| 127 | CDK1 | na |  |  | 2921 | 1.040 | 0.6570 | Yes |
| 128 | CHEK1 | na |  |  | 2954 | 1.034 | 0.6598 | Yes |
| 129 | MYBL2 | na |  |  | 3098 | 1.007 | 0.6567 | Yes |
| 130 | RPA2 | na |  |  | 3128 | 1.001 | 0.6596 | Yes |
| 131 | MMS22L | na |  |  | 3165 | 0.995 | 0.6621 | Yes |
| 132 | MCM5 | na |  |  | 3202 | 0.986 | 0.6645 | Yes |
| 133 | POLD3 | na |  |  | 3244 | 0.979 | 0.6667 | Yes |
| 134 | SYNCRIP | na |  |  | 3486 | 0.939 | 0.6581 | Yes |
| 135 | DCK | na |  |  | 3495 | 0.937 | 0.6618 | Yes |
| 136 | GSPT1 | na |  |  | 3557 | 0.928 | 0.6627 | Yes |
| 137 | SHMT1 | na |  |  | 3578 | 0.924 | 0.6657 | Yes |
| 138 | NASP | na |  |  | 3598 | 0.921 | 0.6687 | Yes |
| 139 | SMC1A | na |  |  | 3638 | 0.915 | 0.6707 | Yes |
| 140 | MSH2 | na |  |  | 3641 | 0.915 | 0.6746 | Yes |
| 141 | DUT | na |  |  | 3726 | 0.900 | 0.6741 | Yes |
| 142 | CIT | na |  |  | 3837 | 0.878 | 0.6722 | Yes |
| 143 | MYC | na |  |  | 3907 | 0.866 | 0.6724 | Yes |
| 144 | MLH1 | na |  |  | 3984 | 0.853 | 0.6721 | Yes |
| 145 | POLD2 | na |  |  | 3987 | 0.852 | 0.6757 | Yes |
| 146 | WEE1 | na |  |  | 3991 | 0.851 | 0.6793 | Yes |
| 147 | H2AFZ | na |  |  | 4017 | 0.848 | 0.6817 | Yes |
| 148 | RAD21 | na |  |  | 4041 | 0.844 | 0.6842 | Yes |
| 149 | ASF1B | na |  |  | 4346 | 0.801 | 0.6717 | No |
| 150 | DCLRE1B | na |  |  | 4652 | 0.756 | 0.6590 | No |
| 151 | STMN1 | na |  |  | 4739 | 0.740 | 0.6577 | No |
| 152 | CDK4 | na |  |  | 4801 | 0.730 | 0.6577 | No |
| 153 | SLBP | na |  |  | 4848 | 0.723 | 0.6585 | No |
| 154 | ILF3 | na |  |  | 4902 | 0.718 | 0.6588 | No |
| 155 | PDS5B | na |  |  | 4949 | 0.714 | 0.6596 | No |
| 156 | LIG1 | na |  |  | 5038 | 0.702 | 0.6580 | No |
| 157 | PTTG1 | na |  |  | 5055 | 0.700 | 0.6602 | No |
| 158 | SPC25 | na |  |  | 5107 | 0.692 | 0.6606 | No |
| 159 | CCNB2 | na |  |  | 5148 | 0.685 | 0.6615 | No |
| 160 | BUB1B | na |  |  | 5225 | 0.675 | 0.6605 | No |
| 161 | NAP1L1 | na |  |  | 5643 | 0.619 | 0.6412 | No |
| 162 | CTCF | na |  |  | 5690 | 0.611 | 0.6415 | No |
| 163 | EED | na |  |  | 5867 | 0.589 | 0.6348 | No |
| 164 | SMC3 | na |  |  | 5872 | 0.588 | 0.6372 | No |
| 165 | PSMC3IP | na |  |  | 5935 | 0.579 | 0.6365 | No |
| 166 | LUC7L3 | na |  |  | 6072 | 0.561 | 0.6318 | No |
| 167 | RAD50 | na |  |  | 6079 | 0.560 | 0.6339 | No |
| 168 | H2AFX | na |  |  | 6160 | 0.547 | 0.6321 | No |
| 169 | PRDX4 | na |  |  | 6214 | 0.538 | 0.6317 | No |
| 170 | CBX5 | na |  |  | 6221 | 0.537 | 0.6337 | No |
| 171 | RPA3 | na |  |  | 6282 | 0.530 | 0.6329 | No |
| 172 | CKS2 | na |  |  | 6345 | 0.522 | 0.6319 | No |
| 173 | RFC1 | na |  |  | 6554 | 0.495 | 0.6232 | No |
| 174 | RAD1 | na |  |  | 6559 | 0.495 | 0.6251 | No |
| 175 | SPC24 | na |  |  | 7205 | 0.421 | 0.5931 | No |
| 176 | HNRNPD | na |  |  | 7535 | 0.381 | 0.5774 | No |
| 177 | PSIP1 | na |  |  | 8404 | 0.284 | 0.5330 | No |
| 178 | TUBG1 | na |  |  | 8708 | 0.245 | 0.5181 | No |
| 179 | WDR90 | na |  |  | 9707 | 0.136 | 0.4662 | No |
| 180 | PPM1D | na |  |  | 10558 | 0.045 | 0.4217 | No |
| 181 | CDC25B | na |  |  | 12061 | -0.134 | 0.3433 | No |
| 182 | HUS1 | na |  |  | 12612 | -0.201 | 0.3152 | No |
| 183 | CDKN1B | na |  |  | 13864 | -0.360 | 0.2510 | No |
| 184 | MXD3 | na |  |  | 14093 | -0.391 | 0.2407 | No |
| 185 | TP53 | na |  |  | 14267 | -0.417 | 0.2334 | No |
| 186 | CHEK2 | na |  |  | 14539 | -0.450 | 0.2211 | No |
| 187 | POP7 | na |  |  | 14581 | -0.460 | 0.2210 | No |
| 188 | PMS2 | na |  |  | 14775 | -0.492 | 0.2130 | No |
| 189 | NAA38 | na |  |  | 15070 | -0.525 | 0.1998 | No |
| 190 | CDKN1A | na |  |  | 15950 | -0.678 | 0.1566 | No |
| 191 | PAN2 | na |  |  | 18269 | -1.473 | 0.0411 | No |
| 192 | HMGB3 | na |  |  | 18612 | -1.764 | 0.0308 | No |
Table: GSEA details [plain text format]

  

Fig 2: HALLMARK\_E2F\_TARGETS      
 Blue-Pink O' Gram in the Space of the Analyzed GeneSet

  

Fig 3: HALLMARK\_E2F\_TARGETS: Random ES distribution      
 Gene set null distribution of ES for **HALLMARK\_E2F\_TARGETS**
